# The solvent chosen for the manufacturing of electrospun polycaprolactone scaffolds influences cell behavior of lung cancer cells

**DOI:** 10.1038/s41598-022-23655-2

**Published:** 2022-11-14

**Authors:** Emma Polonio-Alcalá, Enric Casanova-Batlle, Teresa Puig, Joaquim Ciurana

**Affiliations:** 1 Product, Process and Production Engineering Research Group (GREP), Department of Mechanical Engineering and Industrial Construction, University of Girona, Girona, Spain; 2 New Therapeutic Targets Laboratory (TargetsLab)-Oncology Unit, Department of Medical Sciences, University of Girona, Girona, Spain

**Keywords:** Lung cancer, Biomaterials

## Abstract

The development of a trustworthy in vitro lung cancer model is essential to better understand the illness, find novel biomarkers, and establish new treatments. Polycaprolactone (PCL) electrospun nanofibers are a cost-effective and ECM-like approach for 3D cell culture. However, the solvent used to prepare the polymer solution has a significant impact on the fiber morphology and, consequently, on the cell behavior. Hence, the present study evaluated the effect of the solvent employed in the manufacturing on the physical properties of 15%-PCL electrospun scaffolds and consequently, on cell behavior of NCI-H1975 lung adenocarcinoma cells. Five solvents mixtures (acetic acid, acetic acid-formic acid (3:1, v/v), acetone, chloroform-ethanol (7:3, v/v), and chloroform-dichloromethane (7:3, v/v)) were tested. The highest cell viability ($\overline{x }$ = 33.4%) was found for cells cultured on chloroform-ethanol (7:3) PCL scaffolds. Chloroform-dichloromethane (7:3) PCL scaffolds exhibited a roughness that enhanced the quality of electrospun filament, in terms of cell viability. Our findings highlighted the influence of the solvent on fiber morphology and protein adsorption capacity of nanofilaments. Consequently, these features directly affected cell attachment, morphology, and viability.

## Introduction

Lung cancer is the second most incident cancer and the main cause of cancer-related death among both sexes worldwide, accounting for approximately 2.2 million of new cases and 1.8 million of deceases each year^1^. The most prevalent subtype is non-small cell lung cancer (NSCLC), and around 40% of cases are diagnosed as adenocarcinoma^2^. Almost 60% of patients are detected at advanced stage of the illness when the traditional treatment exhibits a response rate of about 25% and the surgical intervention is not feasible^3,4^. Moreover, several resistant mechanisms to targeted therapies have been described leading to lethal secondary tumors^5,6^ Therefore, the development of a trustworthy lung cancer model is necessary to better understand this aggressive disease.

The two-dimensional (2D) cell culture model is a well-established methodology employed in cancer research. Nonetheless, flat surfaces do not completely reproduce the tumor microenvironment. In physiological conditions, cells are surrounded by the extracellular matrix (ECM), which plays a key role in certain cellular processes, such as drug response and differentiation. Hence, monolayer culture changes cell behavior (i.e. cell proliferation, genetic expression, or protein regulation), apical-basal polarity, nutrients and oxygen distribution, and soluble gradients^7^. Consequently, several three-dimensional (3D) cell culture systems have been investigated to mimic the tissue environment providing a spatial distribution that modifies cell–cell and cell–matrix interactions, morphology, adhesion, alignment, and migration^8^. As a result, 3D cell culture provides a more accurate cell responses and decreases the need for animal trials^9^.

Nanofibers manufactured using electrospinning technique exhibit a filament size similar to ECM and a high surface area-to-volume ratio allowing cell attachment^10^. Polycaprolactone (PCL) is usually chosen to manufacture electrospun scaffolds, which have been proved to be a useful 3D cell culture for lung cancer stem cell population^11^. This synthetic polymer is suitable for biomedical engineering and cell culture applications because its low melting temperature, biocompatibility, long-term biodegradability, bioresorbability, and inexpensive price^12,13^.

The potential of the electrospinning technique to define scaffold characteristics relies on the intrinsic features of the solution such as polymer concentration, viscosity, or conductivity, the control of the manufacturing parameters (i.e., voltage, distance between needle and collector, or flow rate), and the environmental conditions (i.e., temperature and humidity)^14^. Even though these parameters have been studied for their influence on fiber morphology^15–17^, few studies have related the synergy of these parameters with cell behavior^18,19^. Moreover, there is a lack of knowledge on how the solvent used for the solution can affect the fiber morphology and, thus, cell behavior of lung cancer cells. Thus, understanding how to control the fiber morphology, which provides the relationship to cell behavior, by simply modifying the solvent chosen to elaborate the solution, is very valuable when designing models for 3D lung cancer cell culture.

For this purpose, 15%-PCL electrospun scaffolds were manufactured using five solvent solutions: acetic acid, acetic acid-formic acid (3:1, volume/volume), acetone, chloroform-ethanol (7:3, volume/volume), and chloroform-dichloromethane (7:3, volume/volume). The main aim of this study delved into the influence of the solvent of interest in physical changes in the nanofiber and relate it to the impact on cell morphology and viability of lung adenocarcinoma cells, in a direct line.

## Results

### Microstructure of PCL scaffolds

**Figure 1:**
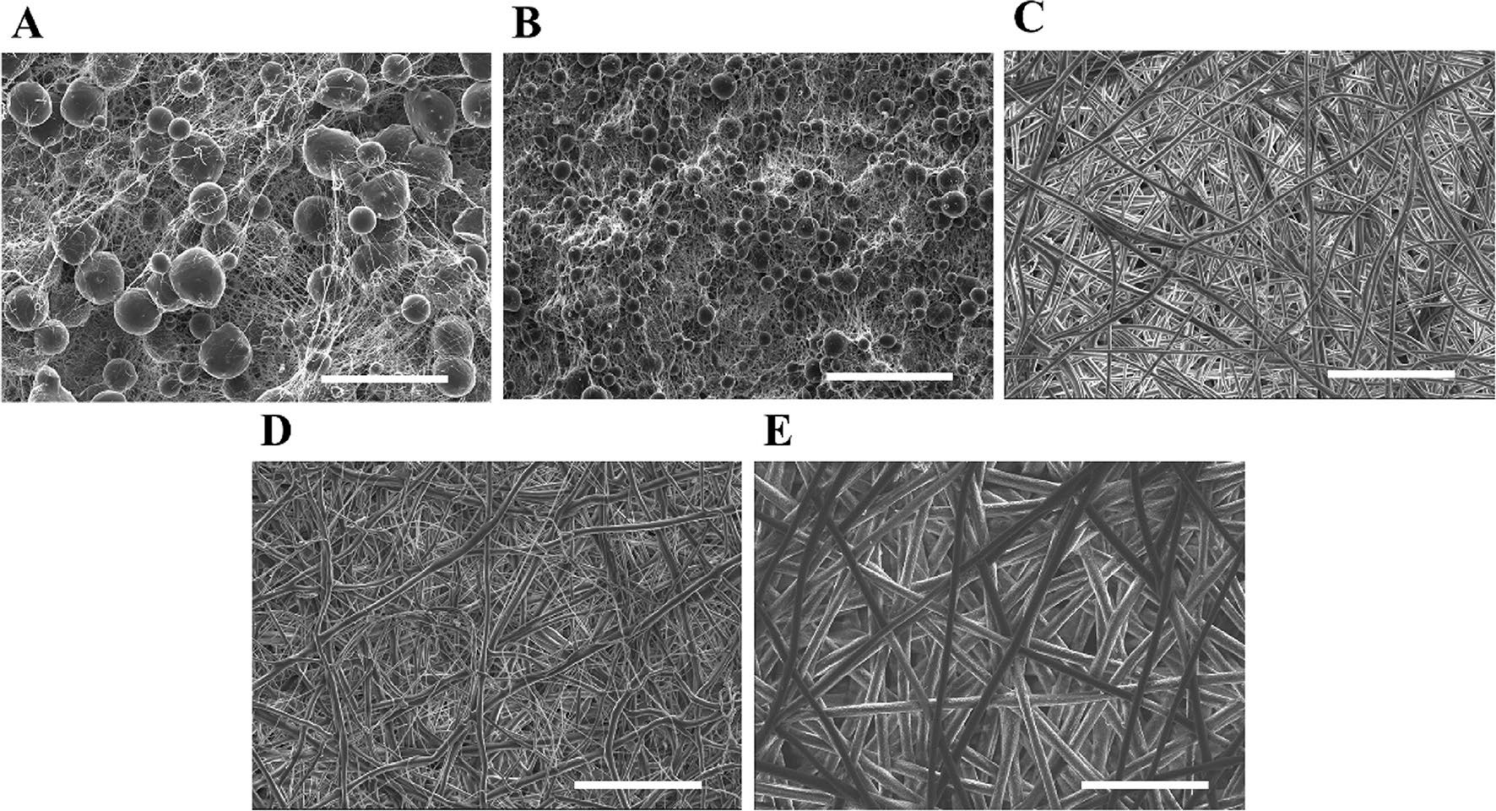
Scanning electronic microscopy (SEM) images from (**A**) AA-PCL scaffolds, (**B**) AA-FA-PCL scaffolds, (**C**) Ace-PCL scaffolds, (**D**) Chl-EtOH-PCL scaffolds, and (**E**) Chl-DCM-PCL scaffolds (scale bar 60 μm).

The microarchitecture of PCL scaffolds was visualized by SEM (Fig. 1) to determine their pore area, porosity, and fiber diameter (FD) (Table 1).

**Table 1:** Pore area, porosity and fiber diameter of PCL scaffolds.

	Pore area (μm^2^)	Porosity (%)	Fiber diameter (μm)
AA	4.19 ± 1.62	21.41 ± 2.94	0.16 ± 0.03
AA-FA	3.88 ± 1.37	15.43 ± 1.76	0.17 ± 0.03
Acetone	299.71 ± 78.54*^$^	56.01 ± 2.76***^$$$^	2.96 ± 0.61***^$$$^
Chl-EtOH	100.13 ± 29.70^#^	48.79 ± 1.63***^$$$^	1.68 ± 0.44**^$$##^
Chl-DCM	592.38 ± 170.40	64.65 ± 3.07***^$$$ℲℲ^	5.97 ± 0.41***^$$$###ℲℲℲ^

Levels of statistically significance are indicated as *(p < 0.050), **(p < 0.010), and ***(p < 0.001). The symbol * indicates the comparison with AA-PCL scaffolds, $ indicates the comparison with AA-FA-PCL-scaffolds, # indicates the comparison with Ace-PCL scaffolds, and Ⅎ indicates the comparison with Chl-EtOH-PCL-scaffolds.

Two subtypes of PCL matrices were shown according to the solvent chosen for the manufacturing process. On the one hand, the employment of AA and AA-FA resulted in very dense 3D meshes. Consequently, these PCL supports exhibited the lowest pore area, being statistically significant compared to the Ace-PCL ones. These 3D platforms also showed a significantly lower porosity and FD compared to Ace-, Chl-EtOH-, and Chl-DCM-PCL ones. Non-filamentous structures (beads) were observed in AA- and AA-FA-PCL nanofibers.

On the other hand, scaffolds produced from the mixtures of PCL and Ace, Chl-EtOH, and Chl-DCM exhibited thicker fibers, on the micrometer scale. No beads were found in the SEM images in these PCL structures. Overall, Chl-DCM-PCL meshes displayed the highest pore area, porosity, and FD, being statistically significant in the FD. Certain roughness was observed in the nanofibers manufactured using Chl-DCM.

### Viscoelastic and Structural Behavior of PCL scaffolds

**Figure 2:**
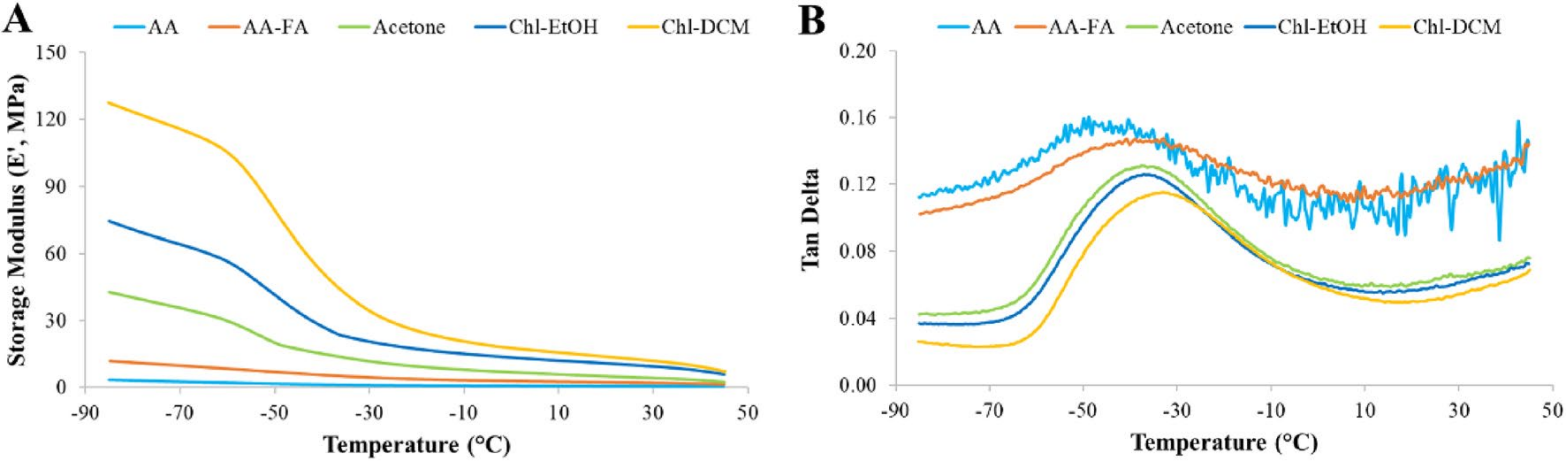
(**A**) Storage modulus (E′) and (**B**) Tan Delta curve obtained by dynamic mechanical analysis (DMA) of PCL scaffolds.

The viscoelastic and structural properties of PCL matrices were revealed by DMA (Fig. 2).

The stiffness of 3D supports was evaluated through the Storage modulus (E′) (Fig. 2A). At physiological temperature (37 °C), E′ value was 0.48 MPa, 1.69 MPa, 3.60 MPa, 8.04 MPa, and 10.12 MPa for AA-, AA-FA-, Ace-, Chl-EtOH, and Chl-DCM-PCL platforms, respectively. The AA-PCL scaffolds were approximately 20 times softer than the Chl-DCM-PCL ones.

The glass transition temperature (T_g_) was obtained by the Tan Delta curve (Fig. 2B), which was very similar for all 3D structures. T_g_ value was −35.27 °C, −33.06 °C, −37.59 °C, −37.11 °C, and −33.18 °C for AA-, AA-FA-, Ace-, Chl-EtOH, and Chl-DCM-PCL matrices, respectively.

### The influence of sterilization procedure and medium soaking

The weight degradation rate was evaluated to examine whether the sterilization process and medium immersion modified PCL meshes (Supplementary Fig. 1).

As a consequence of the sterilization procedure, 3D supports increased approximately 3–6% of their weight. Significant differences were found in AA-, AA-FA, and Ace-PCL platforms between their weight before and after the process (AA: *p* = 0.043; AA-FA: *p* = 0.031; Ace: *p* = 0.037). Additionally, no changes were observed in the weight due to the medium soaking throughout 28 days.

### Adhesion and morphology of cells cultured on PCL scaffolds

**Figure 3:**
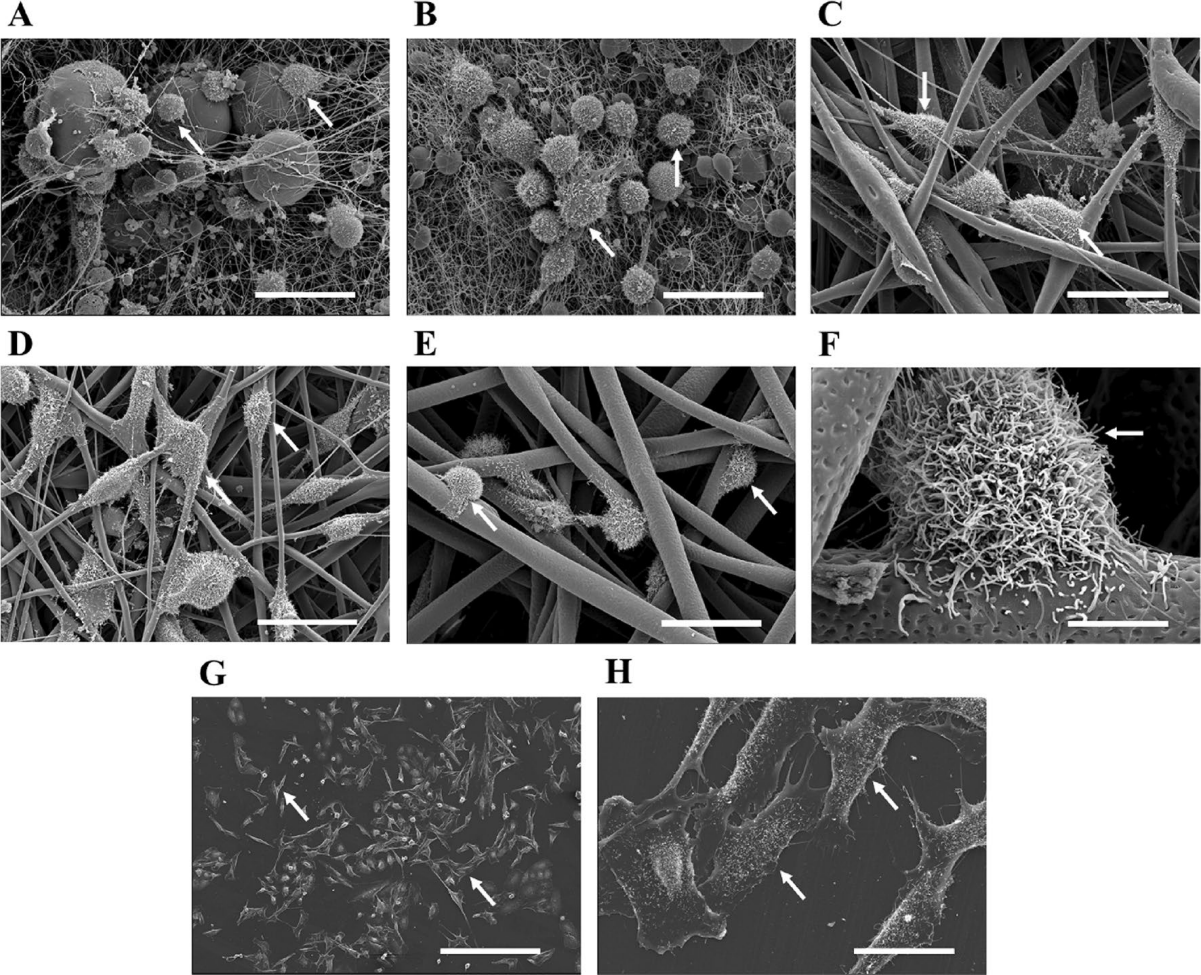
Scanning electronic microscopy (SEM) images from NCI-H1975 cells attached to (**A**) AA-PCL scaffolds, (**B**) AA-FA-PCL scaffolds, (**C**) Ace-PCL scaffolds, (**D**) Chl-EtOH-PCL scaffolds, and (**E**) Chl-DCM-PCL scaffolds (scale bar: 30 μm). (**F**) SEM picture from a NCI-H1975 cell attached to a Chl-DCM-PCL nanofiber due to roughness (scale bar 6 μm). (**G)** SEM picture from NCI-H1975 cells attached to a monolayer (scale bar 300 μm). (**H)** SEM picture from NCI-H1975 cells attached to a monolayer (scale bar 30 μm). Representative cells are indicated by arrows.

NCI-H1975 cell line was cultured on PCL scaffolds for 3 and 6 days and displayed using SEM to investigate the interaction between lung adenocarcinoma cells and the manufactured nanofibers (Fig. 3).

AA- and AA-FA-PCL structures showed thin filaments and pores smaller than cell diameter (Fig. 3A,B). Consequently, cells either adhered to the beads or used the fibers as a network forming a rounded morphology. However, Ace, Chl-EtOH, and Chl-DCM-PCL matrices exhibited thicker filaments which allow cell attachment and wrapping to the fibers (Fig. 3C–E). Cell intercommunication in 3D cell culture was facilitated by these types of meshes since cells could interact with each other through their cilia. These interactions were more abundant in Ace- and Chl-EtOH-PCL supports, which showed ideal spatial distance due to their pore area and porosity. Chl-DCM-PCL nanofibers demonstrated certain roughness, which seemed to be employed by the cells to better attach to the filament (Fig. 3F).

**Figure 4:**
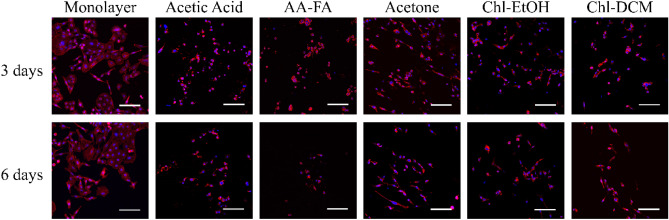
Pictures of NCI-H1975 cells cultured on monolayer and PCL scaffolds for 3 and 6 days displayed by confocal laser scanning microscope (CLSM) at a magnification of ×200 (scale bars 100 µm). Actin cytoskeleton was stained with rhodamine-phalloidin (red) and nucleus with DAPI (blue).

The stained nucleus and cytoskeleton of NCI-H1975 cells seeded on PCL platforms for 3 and 6 days were also visualized by CLSM (Fig. 4) to study the circularity (Eq. 1) of nucleus and cytoplasm of cells (Table 2).

**Table 2:** Circularity of nucleus and cytoplasm of NCI-H1975 cells cultured on PCL scaffolds for 3 and 6 days.

	Nucleus		Cytoplasm	
	3 days	6 days	3 days	6 days
2D	0.82 ± 0.03	0.85 ± 0.03	0.50 ± 0.05	0.54 ± 0.06
AA	0.89 ± 0.02***	0.90 ± 0.02***	0.79 ± 0.05***	0.75 ± 0.05***
AA-FA	0.90 ± 0.02***	0.90 ± 0.02**	0.80 ± 0.05***	0.76 ± 0.06***
Acetone	0.83 ± 0.03^$$$###^	0.83 ± 0.04^$$$###^	0.52 ± 0.06^$$$###^	0.55 ± 0.06^$$$###^
Chl-EtOH	0.87 ± 0.02***^##ℲℲℲ^	0.84 ± 0.03^$$$###^	0.59 ± 0.07**^$$$###^	0.53 ± 0.06^$$$###^
Chl-DCM	0.84 ± 0.03*^$$$###^	0.81 ± 0.04*^$$$###^	0.60 ± 0.07**^$$$###Ⅎ^	0.54 ± 0.07^$$$###^

Value equal to 1 means a circle and 0 no-circle. Levels of statistically significance are indicated as *(p < 0.050), **(p < 0.010), and ***(p < 0.001). The symbol * indicates the comparison with monolayer, $ indicates the comparison with AA-PCL scaffolds, # indicates the comparison with AA-FA-PCL scaffolds, and Ⅎ indicates the comparison with Ace-PCL scaffolds.

Two cell shapes were showed in monolayer cell culture: a round population, which was predominant, but there was also an extremely elongated population. Moreover, different morphologies were noticed when cells were cultured on 3D structures, which was confirmed by SEM images (Fig. 3).

On the one hand, cells seeded on AA- and AA-FA-PCL scaffolds exhibited a rounded shape. Their nucleus and cytoplasm were significantly more circular than cells grown on 2D.

On the other hand, cells cultured on Ace-, Chl-EtOH-, and Chl-DCM-PCL matrices were more elongated. Their nucleus and cytoplasm were significantly less circular in contrast to cells seeded on AA- and AA-FA-PCL meshes. Furthermore, nucleus and cytoplasm of cells grown on Chl-EtOH-PCL platforms for 3 days and Chl-DCM-PCL ones for 3 and 6 days were less circular than on monolayer cell culture, in a significantly way.

### Protein adsorption and cell viability on PCL scaffolds

**Figure 5:**
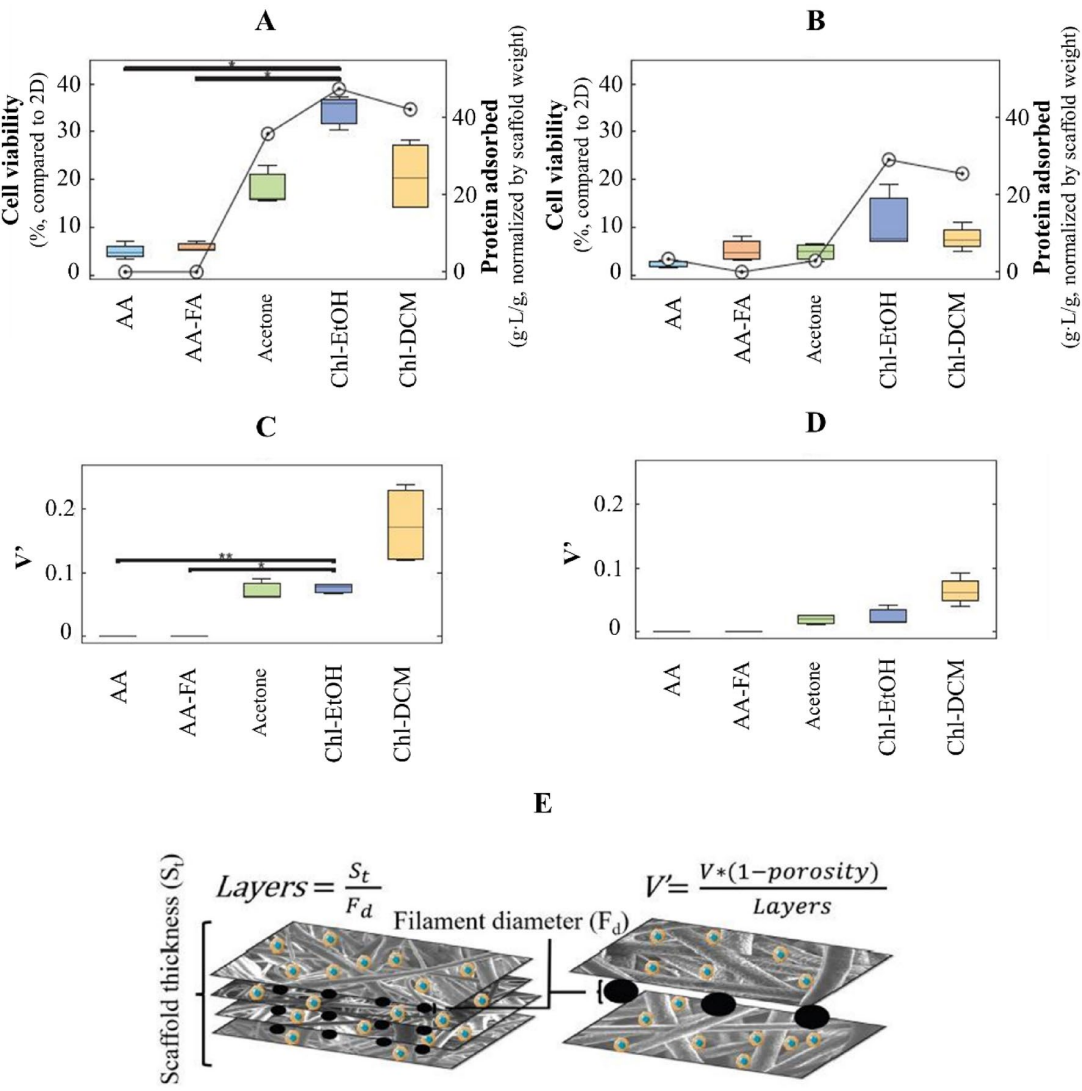
Cell viability and protein adsorption of NCI-H1975 cells cultured on PCL scaffolds for (**A**) 3 and (**B**) 6 days. Cell viability is represented in boxplot form on the left axis. Protein adsorption was normalized by the scaffold weight and is shown as mean with circles on the right axis. Cell viability per fiber of NCI-H1975 cells cultured on PCL scaffolds for (**C**) 3 and (**D**) 6 days. (**E**) Cell viability per fiber (V′). The cell viability was normalized by the porosity and layers of the scaffold to obtain a parameter which represents the cell viability on fibers of each layer.

The cell viability rate of NCI-H1975 cells cultured on PCL scaffolds for 3 and 6 days was evaluated by MTT assay (Fig. 5A,B). Overall, cells seeded on 3D structures showed lower viability than on monolayer, both at 3 and 6 days. The cell viability on AA- and AA-FA-PCL matrices was very poor, less than 10%. Additionally, cells cultured on Ace- and Chl-DCM-PCL meshes for 3 days exhibited a similar viability rate ($\overline{x }$ = 18.1% and $\overline{x }$ = 20.7%, respectively). Concretely, the highest cell viability rate was observed in Chl-EtOH-PCL supports ($\overline{x }$ = 33.4%), being statistically significant in comparison with AA- and AA-FA-PCL ones. Nevertheless, the cell viability on Ace-, Chl-EtOH-, and Chl-DCM-PCL platforms decreased dramatically after 6 days of culture, with values similar to AA- and AA-FA-PCL ones.

The protein adsorption capacity in the surface of nanofibers was also investigated after 3 and 6 days of incubation with medium (Fig. 5A,B). The parameter was normalized by scaffold weight. The results obtained highlighted that the protein adsorbed by PCL structures followed the same trend as the cell viability, both at 3 and 6 days. While 3D meshes that adsorbed proteins exhibited notable cell viability, those nanofibers that demonstrated values similar to 0 g/L, the viability rate was extremely low. Hence, the greatest protein adsorption corresponded to the highest viability rate, which was found in Chl-EtOH matrices. Statistically significant differences were obtained by ANOVA test among the different PCL scaffold types for both conditions (*p* = 3.850 × 10^–4^ for 3 days; *p* = 0.021 for 6 days). Furthermore, protein adsorption was lower after 6 days of incubation than 3 days. Concretely, approximately 40% less protein adsorption for Chl-EtOH- and Chl-DCM-PCL supports and more than 90% for Ace-PCL ones.

Although the cell viability rate allowed the researchers to have a global view of cell behavior, it did not accurately compare cell viability into the nanofibers. In order to better represent this feature, the cell viability per fiber (V’) was defined with the aim to determine the viability in the habitable surface area of a layer of PCL scaffold (Fig. 5E). Interestingly, the greatest V’ was observed in cells seeded on Chl-DCM-PCL supports (x̄ = 0.18% for 3 days; x̄ = 0.06% for 6 days), followed by Chl-EtOH- and Ace-PCL ones, both at 3 and 6 days (Fig. 5C,D). V′ value on AA- and AA-FA was practically zero. Statistically significant differences were found between V′ value of cells grown on Chl-EtOH-PCL platforms and AA- and AA-FA-PCL ones.

## Discussion

Lung cancer is very aggressive and patients show a poor prognosis due to the acquisition of resistance to treatments and the late detection of the disease^3,5,6^. A reliable in vitro lung cancer model must be developed in order to better understand the illness, find new biomarkers, and develop novel therapies^20,21^. Electrospinning is an alternative approach of manufacturing cost-effective ECM-resembling scaffolds for 3D cell culture^10^. The interesting features of PCL and its approval by the Food and Drug Administration (FDA) have led to the increased use of this synthetic polymer in biomedical applications in recent years^12,13^. The few studies focused on the role of the solvent concluded that it directly influences the FD and the presence of beads^15–17^. Other investigations have revealed alterations in cell behavior due to filament morphology^18,19^. To the best of our knowledge, only Guarino and collegues studied, in a direct line, the impact of the solvent on the resulting nanofibers and thus, on cell behavior of a human mesenchymal stem cell line^22^. Additionally, each cell type demands specific 3D cell culture conditions^23^. Therefore, for the first time, the effect of the solvent on the manufacturing and physical properties of 15%-PCL electrospun scaffolds were evaluated, and consequently, on cell behavior of NCI-H1975 lung adenocarcinoma cells. Five solvents or solvent mixtures were selected to prepare the PCL solution: AA, AA-FA (3:1), Ace, Chl-EtOH (7:3), and Chl-DCM (7:3). As far as our knowledge, our study introduces, for the first time, the solvent mixture Chl-DCM (7:3) to dissolve PCL for electrospinning.

Viscosity and conductivity (Table 3) of the solutions are directly related to the FD. Concretely, the FD was thicker by increasing the viscosity^24^. Nezarati et al. demonstrated that viscosity had a more important role in filament morphology than polymer concentration. Their study revealed that beaded fibers appeared at low viscosity, uniform filaments at an intermediate viscosity, and larger fibers at high viscosity^25^. Our results are in agreement with the literature. Chl-DCM-PCL solution exhibited an intermediate viscosity and its filaments were the most uniform. Furthermore, the FD decreases with the increasing conductivity of the solution^26,27^. Concretely, Chl-EtOH- and Chl-DCM-PCL solution had similar viscosities, however the conductivity of Chl-EtOH-PCL solution was higher than Chl-DCM-PCL.

**Table 3:** Viscosity and conductivity of PCL solutions.

Solvent selected for PCL solution	Viscosity (mPa s)	Conductivity (µS/cm^2^)
AA	341.5 ± 9.1	2.15 ± 0.6
AA-FA	185.5 ± 8.9	3.78 ± 0.3
Ace	549.2 ± 46.4	0.25 ± 0.2
Chl-EtOH	487.5 ± 60.0	0.07 ± 0.001
Chl-DCM	485.7 ± 27.4	0.02 ± 0.001

FD, porosity, and pore area (Table 1) were obtained through the analysis of SEM images (Fig. 1). The average FD of Chl-EtOH-PCL scaffolds is consistent with the literature^28^. However, differences were found for AA-, AA-FA-, and Ace-PCL structures. Ace- and AA-FA-PCL nanofibers showed a FD of approximately 3 μm and 170 nm, respectively, while other investigations exhibited thinner filaments for Ace-PCL matrices, about 500–700 nm^29,30^, and thicker fibers for AA-FA-PCL meshes, around 600 nm^31^. Ferreira et al. reported that 14–17% PCL-AA solutions did not have adequate viscosity to be electrospun^32^, whereas our study demonstrated completely opposite findings. These differences could be a result of unequal setup settings, such as the relative humidity^25^. Qin et al. described nanofibers from 15%-PCL dissolved with DCM or Chl depicted an average FD of approximately 4.5 µm and 2 µm, respectively^30^. Moreover, the FD of DCM-PCL supports exhibited a large standard deviation (≈ 2 µm). Our results demonstrated thicker filaments by mixing both solvents, but the standard deviation was reduced compared to DCM-PCL platforms. In addition, the authors mentioned that both solvents caused roughness in the fibers due to their high volatility, which it was also observed in Chl-DCM-PCL scaffolds (Fig. 1E). Lawrence and Madihally pointed out that a porosity of ≥ 90% is recommended for an optimal transport of nutrients and waste products, cell adhesion, migration and ingrowth into the scaffolds, and ECM regeneration and deposition^33^. None of the PCL structures of our study demonstrated such large porosity (Table 1). Nevertheless, Ace-, Chl-EtOH-, and Chl-DCM-PCL meshes displayed superior cell adhesion to the fibers (Figs. 3, 4) and higher cell viability in comparison with AA- and AA-FA-PCL supports, which shown a lower porosity in contrast to the other PCL scaffolds (Fig. 5A,B). Moreover, pore area also plays a key role for cell culture. The infiltration of cells into the scaffold is limited due to a too small pore area, thus the 3D culture becomes a 2D culture with roughness^34^. This is the case with AA- and AA-FA-PCL scaffolds. Cells could penetrate between the fibers meshwork and remained on the scaffold surface (Fig. 3A,B). In contrast, the pore area of Ace-, Chl-DCM- and Chl-EtOH-PCL scaffolds were large enough for cell infiltration and, consequently, 3D cell culture (Fig. 3C–E). Furthermore, NCI-H1975 cells exhibited an enhanced cell proliferation, which is in agreement with the literature^35^.

The appropriate stiffness of 3D platforms is crucial for cell attachment, morphology, or viability^36^. The E′ value for the primary element of 2D cell culture plates, polystyrene, is approximately 2100 MPa, while for a healthy lung tissue is about 1.4 kPa^37,38^. Hence, PCL structures were softer than 2D plates but stiffer than lung tissue, fluctuating from 0.48 to 10.12 MPa at 37 °C. Qin et al. proved that there were small temperature variations among different PCL nanofibers depending on the solvent with which the polymer was dissolved^30^, as also revealed in our study (Fig. 2B), ranging from −37.59 to −33.18 °C. However, PCL matrices exhibited a lower Tg than the Tg of PCL material found in the literature, around −60 °C. These differences in Tg could be a consequence of the sterilization process. Actually, the sterilization method, based on overnight ethanol and 30 min of UV light, increased the weight of PCL meshes by 3–6%. Guerra et al. exposed that the use of ethanol 70% for sterilization produced a reduction of approximately 12% in the Mw^39^. Nonetheless, no changes were exhibited in their weight because of the medium immersion for 28 days, which is in agreement with the literature^40^.

NCI-H1975 cells were cultured on PCL scaffolds for 3 and 6 days. Cells seeded on AA- and AA-FA-PCL matrices showed higher circularity than cells grown on 2D and on Ace-, Chl-EtOH-, and Chl-DCM-PCL meshes (Fig. 4 and Table 2). This is probably due to the fact that AA- and AA-FA-PCL scaffolds showed beads in their fibers (Fig. 1A,B), as reported in other studies^29^. Although cell adhesion to all PCL structures was confirmed (Fig. 3), the elongated cell morphology has more focal points for better attachment than the round cell morphology, which has weaker adhesion due to the reduction of these focal points^41^. Both morphologies using electrospun nanofibers have previously been reported in the literature. Rabionet et al. found cell elongation in breast cancer cells cultured on PCL nanofibers^29,42^, whereas other studies used electrospun fibers to develop spheroids^43,44^. Moreover, cells seeded on AA- and AA-FA-PCL supports showed round shape (Fig. 3A,B), and also exhibited the lowest cell viability (Fig. 5A,B). In contrast, other studies pursued round morphology to increase cell viability in human mesenchymal and adipose-derived stem cells^43,44^. Interestingly, different studies reported that lung cancer cells seeded on decellularized lung scaffolds showed a rounded shape^45,46^. However, pathologists reported that tumor cells from patients have an irregular shape, do not form a uniform layer, and have a larger nucleus^47^.

Chl-EtOH-PCL scaffolds demonstrated the highest cell viability and the thinner filaments compared to Ace- and Chl-DCM-PCL matrices (Fig. 5A,B), which is in agreement with the literature^48^. Previous research suggested that some types of cells exposed a better attachment and viability due to the roughness of filament^49^. The second highest viability was found in cells grown on Chl-DCM-PCL structures, although these 3D matrices displayed the highest FD. Thus, the roughness of Chl-DCM-PCL nanofibers produced a more suitable environment for a better cell viability. In contrast, AA- and AA-FA-PCL scaffolds, which had the thinnest filaments with beads, exhibited the lowest cell viability. These results are in agreement with Chen et al., who demonstrated that these artifacts decreased cell adhesion and growth kinetics^50^. Hence, beads are not desired when the aim of the 3D scaffold is cell culture. A novel parameter, V’, to calculate cell viability in the habitable surface of PCL filaments was defined with the aim to indicate their quality (Fig. 5C–E). Interestingly, cells cultured on Chl-DCM-PCL supports demonstrated the highest V′ value, followed by Chl-EtOH- and Ace-PCL ones, respectively. Therefore, V′ value exposes the importance, not only of the FD, but also of the roughness for an enhanced cell viability.

As also shown in Fig. 5A,B, the protein adsorbed by PCL matrices followed the same pattern as cell viability, both at 3 and 6 days. Protein adsorption of a 3D scaffold has a major impact on cell-scaffold interaction influencing cell adhesion and viability^51^. Other studies concluded that the larger surface-to-volume ratio in the nanofibers, the higher protein adsorption capacity^41,52^. For instance, Chl-EtOH-PCL meshes showed a small FD and porosity that maximized surface-to-volume ratio of the overall scaffold providing the maximum adsorbed protein, which also exhibited the highest cell viability. Nonetheless, a considerable decrease of protein adsorption was found after 6 days of incubation due to the excess adsorption after a certain period of time and the protein desorption rate^53^.

Although AA- and AA-FA-PCL matrices showed the formation of beads under the conditions tested in this study, these 3D structures could be optimized. If it is desired to explore these solvents further in subsequent studies, some changes could be performed. For instance, a higher polymer concentration will cause an increase of viscosity, producing more uniform fibers^25^. Another feature would be the environmental conditions since a lower humidity reduce the length of the initial jet, for example^54^. Alternatively, a higher voltage or distance between the needle and the collector would increase the solvent evaporation, preventing the formation of beads^55^.

To sum up, the choice of solvent to prepare the polymer solution has a significant impact on the fiber morphology and, consequently, on the cell behavior of lung adenocarcinoma cells. Our research demonstrated that solvent influenced viscosity and conductivity, properties directly related to FD. At the same time, FD, porosity, pore area, roughness, and stiffness, directly affected cell attachment, morphology, and viability. Our findings also highlighted the strong correlation between the protein adsorption capacity of PCL scaffolds with cell viability. Hence, this study proposes protein adsorption as a cost-effective option to assess the potential of 3D structures, but further investigations are necessary to confirm the association between these parameters. Finally, for the first time, a new parameter, V′, is introduced to calculate the ability of fibers to provide an optimal environment for 3D cell culture. Therefore, this study concludes that the solvent influences cell behavior and, thus, should be considered in every investigation.

## Methods

### Chemicals and reagents

Polycaprolactone (PCL, Mn 80,000 g/mol), dichloromethane (DCM, ≥ 99% (GC)), 3-(4,5-dimethyl-2-thiazolyl)-2,5-diphenyl-2H-tetrazolium bromide (MTT), paraformaldehyde, glutaraldehyde, sodium cacodylate, Triton™ X-100, and bovine serum albumin (BSA) (≥ 98.0%) were purchased from Sigma-Aldrich (St. Louis, MO, USA). Chloroform (stabilized with amylene; ≥ 98% AGR), acetic acid (glacial, 99,8% AGR), formic acid (98% AGR), and ethanol absolute (AGR) were obtained from Labkem, Labbox Labware S.L. (Barcelona, Spain). BSA Fraction V pH for Western blotting (min. 96%) and acetone (min. ≥ 99.8%) were purchased from PanReac AppliChem (Gatersleben, Germany). RPMI-1640 medium, 10,000 U/mL penicillin/streptomycin, phosphate-buffered saline (PBS), and trypsin 10 × were obtained from Lonza (Basilea, Switzerland). Fetal bovine serum (FBS) and l-glutamine 200 mM were purchased from HyClone (Logan, UT, USA). DC Protein Assay was obtained from Bio-Rad (Hercules, CA, USA). Rhodamine-phalloidin was purchased from Cytoskeleton Inc. (Denver, CO, USA) and 4,6-diamidino-2-phenylindole (DAPI) was obtained from BD Pharmingen (Franklin Lakes, NJ, USA).

### Cell line

NCI-H1975 human NSCLC cell line was purchased from the American Type Culture Collection (ATCC; Rockvville, MD, USA). Cells were routinely grown in RPMI-1640 medium supplemented with 10% FBS, 1% l-glutamine, and 50 U/mL penicillin/streptomycin. Cells were kept at 37 °C and 5% CO_2_ atmosphere. They were monitored regularly and also analyzed to be mycoplasma-free.

### The manufacturing of electrospun PCL nanofibers

PCL was dissolved in chloroform-dichloromethane (Chl-DCM) (7:3, volume/volume), chloroform-ethanol (Chl-EtOH) (7:3, volume/volume), acetone (Ace), acetic acid (AA) or acetic acid-formic acid (AA-FA) (3:1, volume/volume) at 15% (weight/volume) for 24 h at 60 °C and under agitation.

The viscosity and electrical conductivity of the solutions (Table 3) were measured with a Myr Serie VR 3000 rotatory viscometer (Viscotech Hispania S.L.; Tarragona, Spain) and an EC-meter basic 30 + conductivity meter (Crison; Barcelona, Spain), respectively.

Scaffolds were manufactured using an electrospinning device (Spraybase, Dublin, Ireland). Polymeric solution was transferred to 20 mL syringe, which was connected through polytetrafluoroethylene tube (inner diameter of 1 mm) to stainless steel 24G needle (inner diameter of 0.55 mm). The machine was set up by the Syringe Pump Pro software (New Era Pump Systems; Farmingdale, NY, USA) (Table 4) and 5 mL of solution were ejected. These values ensured the formation of the Taylor cone during all the process of electrospinning. The resulting structures were kept at room temperature for at least 24 h to ensure a correct solvent evaporation, cut into squares of 2.56 cm^2^, and sterilized as previously described^56^.

**Table 4:** The parameters voltage, flow rate, and distance to collector selected for each PCL solution to conduct the electrospinning procedure.

Solvent selected for PCL solution	Voltage (kV)	Flow rate (mL/h)	Distance to collector (cm)
AA	11	6	14
AA-FA	11	6	11
Ace	8	6	12
Chl-EtOH	8	5	15
Chl-DCM	8	6	20

### Dynamic mechanical analysis (DMA)

The structural and viscoelastic behavior of PCL scaffolds (Table 5) was determined by the DMA using Mettler-Toledo DMA/SDTA861e (Mettler-Toledo; Columbus, OH, USA). DMA was performed at a heating rate of 5 °C from – 85 to 45 °C with 1 Hz of frequency and 50 μm of amplitude at tensile mode.

**Table 5:** The size of samples of PCL scaffolds used for DMA.

	Length (mm)	Width (mm)	Thickness (mm)
AA	5.50	5.18	0.60
AA-FA	5.50	6.80	0.45
Ace	5.50	6.95	0.83
Chl-EtOH	5.50	6.45	0.72
Chl-DCM	5.50	6.74	0.89

### Degradation assay

Scaffolds were weighed by Sartorius ED224S analytical balance (Sartorius, Göttingen, Germany), sterilized, and transferred to non-adherent cell culture 12-well plates (Sarstedt, Nümbrecht, Germany). Supplemented medium was added into each well and kept in the incubator for 3, 6, 14, or 28 days. Afterwards, structures were washed two times with PBS, air-dried, and weighed again. Control samples were directly air-dried after their sterilization.

### Protein adsorption assay

PCL scaffolds were sterilized and put into non-adherent cell culture 12-well plates. They were immersed in 2 mL of supplemented medium and blank samples in PBS, and kept at 37 °C and 5% CO_2_ atmosphere for 3 and 6 days. Afterwards, structures were washed two times with PBS, and placed in new wells to ensure to only analyze proteins attached to PCL scaffolds. Following the manufacturer protocol, DC Protein Assay was performed in order to quantified the amount of protein, which was calculated based on a BSA standard curve. Three aliquots from each well were pipetted into 96-well plate and placed into a microplate reader (Bio-Rad) where absorbance was measured at 700 nm.

### Three-dimensional cell culture

Sterilized scaffolds were placed in non-adherent cell culture 12-well plates, soaked in medium, and kept at incubator for at least 30 min with the aim to promote cell attachment. The pertinent cell density (3 days: 75,000 cells; 6 days: 12,000 cells) was prepared in 50 μL of medium. NCI-H1975 cells were seeded on scaffolds as described elsewhere^57^. Monolayer cell culture was performed as control in adherent cell culture 12-well plates.

### PCL scaffold microstructure and cell attachment observation

Sterilized scaffolds were cut and coated with carbon by K950 turbo evaporator (Emitech, Kent, UK). On the other hand, seeded samples were fixed by 2.5% glutaraldehyde solution (volume/volume) (in 0.1 M sodium cacodylate, pH 7.4), washed in 0.1 M sodium cacodylate and dehydrated in a graded series of ethanol (50, 75, 80, 90, 95, and 100%). Structures were dried using K850 CPD critical point dryer (Emitech), and coated with gold by K950 turbo evaporator (Emitech). Observations were performed by S4100 field emission scanning electron microscopy (SEM; Hitachi, Tokyo, Japan). Images were digitally captured by Quartz PCI software (Quartz, Vancouver, Canada). Surface porosity, fiber diameter and pore area were determined through MATLAB software (MathWorks; Natick, MA, USA). Briefly, the porosity was determined by image segmentation, where the percentage of filament and background of the image was calculated. The filament diameter was measured by calculating the distance of the perpendicular line between the top and bottom end of the filament. The pore area was calculated by manually drawing the shape of the pores on the images. Three filaments and pores were randomly selected from each photo. The beads were not considered in the fiber diameter study.

### Nuclear and cytoplasmic circularity

NCI-H1975 cells were seeded on adherent coverslips (Sarstedt) or PCL scaffolds for 3 and 6 days. Samples were fixed using 4% paraformaldehyde solution (weight/volume), permeated by 0.2% Triton ™ X-100 (volume/volume), blocked by 3% BSA solution (weight/volume), and dyed using rhodamine-phalloidin (1:250) and DAPI (1:1000). Fluorescence was observed under an A1R confocal laser scanning microscope (CLSM; Nikon, Tokyo, Japan). Images were taken through Nikon NIS-Elements AR v4.10 software (Nikon). Nuclear and cytoplasmic circularity was determined using MATLAB software (MathWorks). At least three replicates of each type of PCL scaffold were made at 3 and 6 days. For each replicate, three representative images were taken. Subsequently, a software was implemented to count the number of cells, calculate the sample size knowing the size of the population, and randomly choose the cells from which the perimeter and area were calculated. Then, the circularity was calculated with the following formula:

1 $$Circularity=\frac{4*\pi *area}{{perimeter}^{2}}$$

The equation was designed to give values around 0 for an elongated nucleus or cytoplasm, whereas a perfect circle will give a circularity value of 1.

### Cell proliferation assay

NCI-H1975 cells were seeded into adherent cell culture 12-well plates and PCL scaffolds for 3 and 6 days. Thereafter, PCL samples were washed two times with PBS and transferred to new wells to ensure only attached cells would be analyzed. Finally, the MTT assay was performed as previously described^57^. At least three replicates of each type of PCL scaffold and each time interval were performed.

### Data analysis

The results obtained were confirmed by at least three independent experiments. The statistical analysis was performed using the IBM SPSS software (Version 25.0; SPSS Inc., IL, USA). The data are represented as mean ± standard error of the mean (SE). Parametric data were evaluated by the Student’s t test when comparing two groups or the one‐way analysis of variance (ANOVA) followed by Bonferroni or Tamhane’s T2 post-hoc test for multiple comparisons. Non‐parametric data were analyzed with the Mann–Whitney U tests for non‐normally independent variables or the Kruskal–Wallis test was performed for more than two groups. Levels of significance were established at p < 0.005 and represented as follows: * when p < 0.05, ** when p < 0.01, and *** when p < 0.001.

## Supplementary Information

Supplementary Information.

## Data Availability

All data required to reproduce these findings are available in the manuscript.
