# Supplementary material for: The solvent chosen for the manufacturing of electrospun polycaprolactone scaffolds influences cell behavior of lung cancer cells
Source: Sci Rep. 2022 Nov 14;12:19440. doi: 10.1038/s41598-022-23655-2 (PMC9663546; doi:10.1038/s41598-022-23655-2)
Supplement: Supplementary file 1 — Supplementary Information. [file 41598_2022_23655_MOESM1_ESM.docx]

**Supplementary Images**

**
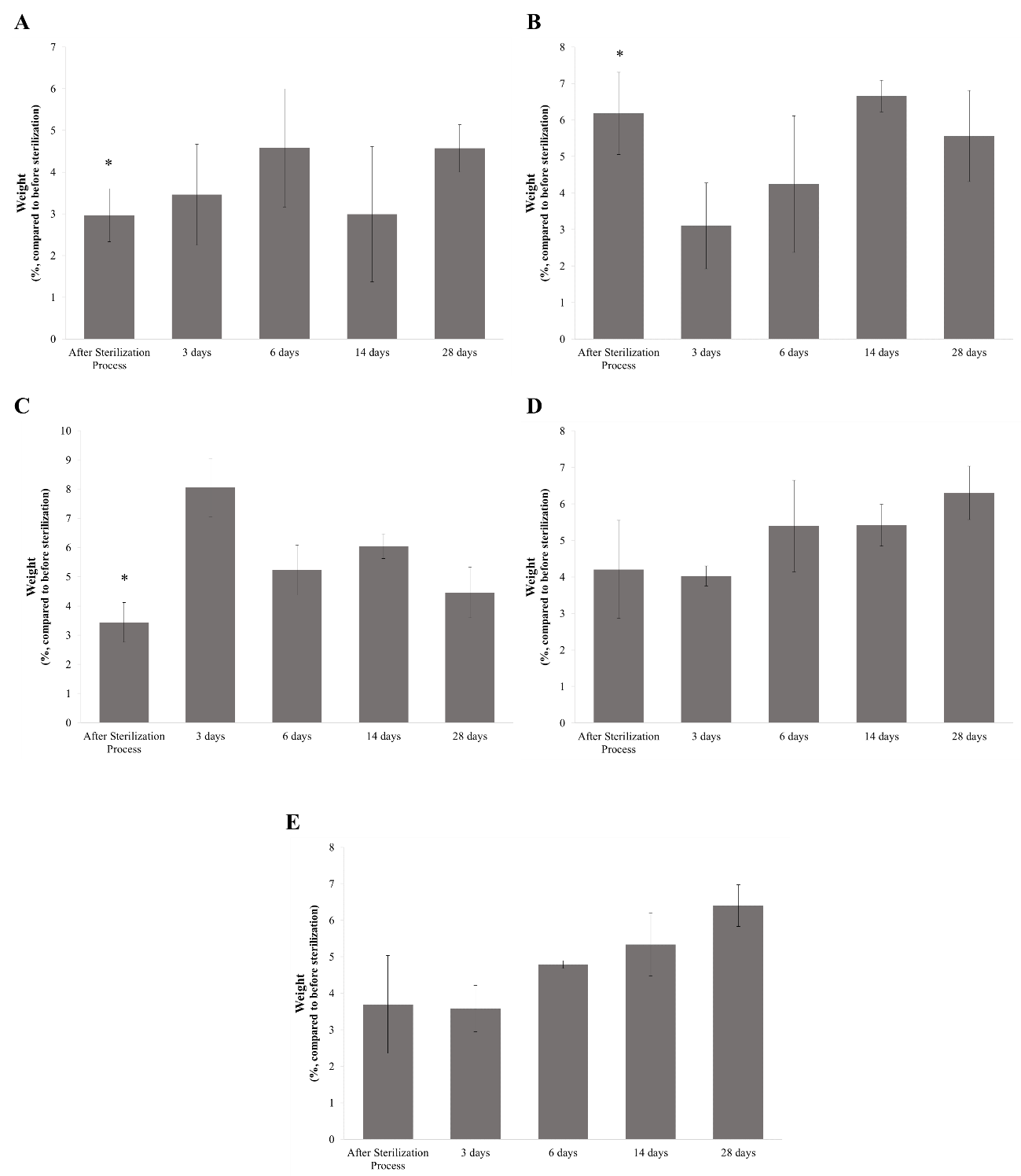
**

**Supplementary Figure 1.** Weight degradation rate of **(A)** Acetic acid, **(B)** Acetic Acid-Formic Acid (3:1), **(C)** Acetone, **(D)** Chloroform-Ethanol (7:3) and **(E)** Chloroform-Dichloromethane (7:3) PCL scaffolds.
